# Development of prediction model of low anterior resection syndrome for colorectal cancer patients after surgery based on machine‐learning technique

**DOI:** 10.1002/cam4.5041

**Published:** 2022-07-28

**Authors:** Ming Jun Huang, Lin Ye, Ke Xin Yu, Jing Liu, Ka Li, Xiao Dong Wang, Ji Ping Li

**Affiliations:** ^1^ West China School of Nursing/Day Surgery Center West China Hospital, Sichuan University Chengdu China; ^2^ West China School of Stomatology Sichuan University Chengdu China; ^3^ Division of Gastrointestinal Surgery, Department of General Surgery West China Hospital, Sichuan University Chengdu China; ^4^ West China Medical School Sichuan University Chengdu China; ^5^ West China School of Nursing/West China Hospital Sichuan University Chengdu China; ^6^ Nursing Department West China Hospital, Sichuan University Chengdu China

**Keywords:** colorectal neoplasm, low anterior resection syndrome, machine learning, prediction model, risk factors

## Abstract

**Background:**

Low anterior resection syndrome (LARS) is a common postoperative complication in patients with colorectal cancer, which seriously affects their postoperative quality of life. At present, the aetiology of LARS is still unclear, but some risk factors have been studied. Accurate prediction and early management of medical intervention are keys to improving the quality of life of such high‐risk patients.

**Objectives:**

Based on machine‐learning methods, this study used the follow‐up results of postoperative patients with colorectal cancer to develop prediction models for LARS and conducted a comparative analysis between the different models.

**Methods:**

A total of 382 patients diagnosed with colorectal cancer and undergoing surgery at West China Hospital from April 2017 to December 2020 were retrospectively selected as the development cohort. Logistic regression, support vector machine, decision tree, random forest and artificial neural network algorithms were used to construct the prediction models of the obtained dataset. The models were internally validated using cross‐validation. The area under the curve and Brier score measures were used to evaluate and compare the differentiation and calibration degrees of the models. The sensitivity, specificity, positive predictive value and negative predictive value of the different models were described for clinical use.

**Results:**

A total of 342 patients were included, the incidence of LARS being 47.4% (162/342) during the six‐month follow‐up. After feature selection, the factors influencing the occurrence of LARS were found to be location, distance, diverting stoma, exsufflation and surgical type. The prediction models based on five machine‐learning methods all showed acceptable performance.

**Conclusions:**

The five models developed based on the machine‐learning methods showed good prediction performance. However, considering the simplicity of clinical use of the model results, the logistic regression model is most recommended. The clinical applicability of these models will also need to be evaluated with external cohort data.

## Graphical abstract

Low anterior resection syndrome (LARS) is a common postoperative complication in patients with colorectal cancer, which seriously affects their postoperative quality of life. We developed LARS prediction models for 382 patients based on 5 different machine learning algorithms. All five models showed good predictive performance and stability and can be considered as an aid to clinical decisions.

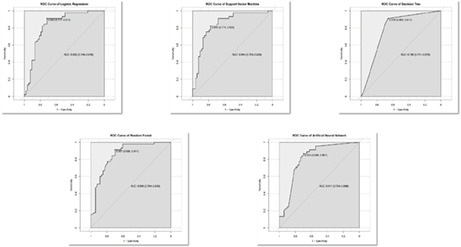

## 1 INTRODUCTION

According to GLOBOCAN 2020, there were more than 1.9 million new cases of colorectal cancer and 935,000 deaths in 2020, accounting for approximately 1/10 cancer cases and deaths.^1^ Colorectal cancer is still the third most common cancer in the world, with the second‐highest death rate, and it is even increasing by 1%–4% per year in high‐income countries such as the United States, Australia and Canada.^1^ With advances in medical technology, the survival rate of colorectal cancer patients has improved significantly over the past 20 years, with its five‐year overall survival rate reaching 59%.^2^ The improvement in surgical techniques, the development of circular stapling devices, and neoadjuvant therapy are helping patients with colorectal cancer avoid permanent stoma. Patients can undergo low anterior resection (LAR) with preservation of the sphincter, thereby reducing the physical and psychological problems associated with a stoma and reducing local recurrence and mortality.^3^, ^4^ However, postoperative intestinal dysfunction often occurs in patients with LAR, seriously affecting their postoperative quality of life.^5^ A large number of patients undergoing anterior resection experience a series of postoperative symptoms, including frequent defecation, urgent defecation, anal incontinence and defecation disorders, collectively known as low anterior resection syndrome (LARS).^6^

LARS is a bowel disorder after sphincter‐preservation rectal resection. In current studies, the incidence of LARS in patients with rectal cancer after surgery is between 30% and 70%.^7^, ^8^, ^9^, ^10^, ^11^, ^12^, ^13^, ^14^, ^15^, ^16^, ^17^ The aetiology of LARS is multi‐factorial—possibly caused by sphincter injury, anorectal physiological changes and pubic neuropathy in the process of anastomosis. LARS affects several aspects of a patients' life, such as physiology, self‐knowledge, emotions and social functions.^18^

Owing to the high incidence of LARS, it is crucial to predict its occurrence in different colorectal cancer patients after surgery. At present, the LARS scoring scale for colorectal cancer developed by Danish scholar Emmertsen in 2012 is often used to evaluate the degree of LARS after surgery,^19^ however, there is currently a lack of LARS prediction tools. Therefore, in this study, multiple machine‐learning algorithms were applied to build predictive models for patients after colorectal surgery. These algorithms could learn complex relationships between features and outcomes, helping medical staff make relevant predictions by learning from the complex and heterogeneous data types. In this study, machine‐learning algorithms were used to develop prediction models for predicting the occurrence of LARS in postoperative patients with colorectal cancer and thus provide an early reference for customised medical programmes.

## 2 METHODS

### 2.1 Clinical data source

Patients undergoing colorectal cancer surgery in West China Hospital of Sichuan University from May 2017 to December 2020 were retrospectively selected as the research subjects. The number of positive events in the sample size is required to be ten times the number of predictors. Referring to previous studies, we considered the incidence of LARS after colorectal cancer surgery to be 70% and assumed a loss to the follow‐up rate of 15%. Based on the electronic medical record system of West China Hospital and Database from Colorectal Cancer, we collected basic patient data, including demographic data (such as gender, age and education level), clinical history (such as hypertension, diabetes and cardiovascular disease history) and clinicopathological data (such as tumour location, tumour size, surgical type and tumour node metastasis stage). All enrolled patients were informed of the study and signed informed consent, the study having been approved by the Hospital Ethics Committee (Grant number: No. 2020 [832]). As a retrospective study, this study was registered in the Chinese Clinical Trial Registry (Registration number: ChiCTR2100048467).

### 2.2 Inclusion and exclusion criteria

Patients included in the study had to have a pathologic diagnosis of colorectal cancer and be at least 18 years old. These patients had not previously undergone colorectal resection before agreeing to participate in the study. Patients with a history of gastrointestinal or other abdominal surgery and colorectal cancer with other malignancies and severe infections were excluded. Patients whose cancer was found to have metastasised or recurred during follow‐up, who were taking drugs that significantly affected intestinal function, and who had their prophylactic stoma open were excluded from the study. Patients with incomplete follow‐up data due to refusal or death were also excluded from the analysis.

### 2.3 Outcome assessment

LARS scores were obtained by telephone or email follow‐up 1, 3 and 6 months post‐operation. The LARS score has a higher specificity and sensitivity to LARS problems than other questionnaires assessing defecation and urinary function such as the Wexner scale and the Kirwan scale. Because it was developed specifically to assess the severity of LARS after low anterior resection and has been validated in multiple centres in several countries, it has good reliability and validity. The patients' symptoms were evaluated using the LARS scoring scale (the Chinese translation version) consisting of five items: exhaust incontinence, defecation incontinence, defecation frequency, sense of tenseness and urgency of defecation; each item was scored quantitatively based on the degree and frequency. The total score could range from 0 to 42: a score of ≤20 indicative of no LARS and thus good intestinal function; a score of 21–29 classified patients as having mild LARS, indicative of light intestinal dysfunction; a score of 30–42 classified patients as having severe LARS, indicative of severe intestinal dysfunction. The cohort study proved that the Chinese version of the LARS scale exhibited good reliability and validity within the Chinese population.^20^ To reduce measurement bias, we set up a separate follow‐up group, all follow‐up personnel being trained and investigated before the study to ensure the credibility of the results. Moreover, the data analyst was unaware of the specific source and encoding of the data.

### 2.4 Model development

#### 2.4.1 Selection of predictive variables

The selection of variables was based on LARS risk factors screened out using several previous retrospective studies and meta‐analyses,^16^, ^17^, ^21^, ^22^ combined with the clinical experience of clinicians. A total of 20 potential risk factors were selected, including patient demographic data, tumour location, tumour size, surgical approach, diverting stoma, neoadjuvant therapy and tumour distance to the dentate line. The definition, naming and encoding of variables are summarised in Table 1.

**TABLE 1:** Variable definition and encoding

Variable	Definition	Encoding
AGE	Age of patient at diagnosis of rectal cancer	–
SEX	–	Male:0 Female:1
BMI	Patient's body mass index	–
CAREER	Type of occupation of the patient	One‐hot encoding
DEGREE	The education level of the patient	Primary school:0 Junior high school:1 High school:2 University degree or above:3
DIABETES	Whether the patient had diabetes before surgery	No:0 Yes:1
HYPERTENSION	Whether the patient had hypertension before surgery	No:0 Yes:1
REPRODUCTIVE DYSFUNCTION	All reproductive system‐related diseases, urological and gynaecological complications present in the patient before surgery	No:0 Yes:1
NEOADJUVANT THERAPY	Whether the patient received neoadjuvant therapy	No:0 Yes:1
CHEMOTHERAPY	Whether the patient received adjuvant chemotherapy during treatment	No:0 Yes:1
DIRECTION	The position of the tumour in the lumen of the rectum	One‐hot encoding
SIZE	Tumour length times width times height (cm^3^)	–
DISTANCE	The distance between the lower margin of the tumour and the dentate line (cm)	–
TNM	TNM pathological staging of colorectal cancer	I:0 II:1 III:2 IV:3
LOCATION	According to the distance of the tumour from the anal margin Lower rectum: below the peritoneum reflexes (0–8 cm) Middle rectum:8–16 cm Upper rectum:16–19 cm Colon	Lower rectum:0 Middle rectum:1 Upper rectum:2 Colon:3
OBSTRUCTION	The severity of intestinal obstruction in the patient No: The patient's bowel lumen was normal and had no symptoms of obstruction Light: The patient had no obvious symptoms of obstruction, but had intestinal stenosis Severe: The patient presented with interstitial abdominal pain, obstructed defecation and intestinal stenosis	No:0 Light:1 Severe:2
SURGICAL TYPE	The type of surgery the patient underwent. Mainly including CAA, CAAN, HAR, LAR, ULAR	One‐hot encoding
ANASTOMOTIC TYPE	Proximal and distal anastomotic type during intestinal reconstruction: End‐to‐end or end‐to‐side	End‐to‐end:0 End‐to‐side:1
CONTRACTED PELVIS	The influence of the degree of pelvic stenosis on the difficulty of operation No: No influence Light: It only affects the operating fluency of the main operator Severe: The main operator should not enter the peritoneal recursion below operation	No:0 Light:1 Severe:2
ADHESIONS OF PELVIC ORGANS	The degree of adhesion between pelvic tissues and organs under abnormal anatomical conditions No: Normal anatomic relation Light: Slight adhesion but does not affect operation Severe: The bowel is distorted by adhesion and needs to be dissociated or removed	No:0 Light:1 Severe:2
EXSUFFLATION	The number of days between the patient's first exhaust and surgery	–
RISK OF LEAKAGE	Risk of postoperative anastomotic leakage (predicted by colo‐anal anastomosis, inflammatory or insomniac constitution, intestinal quality and degree of cancerous edema)	Low:0 High:1
SURGICAL COMORBIDITY	In addition to colorectal cancer, other diseases may require surgical intervention (or invasive procedures)	No:0 Yes:1
DIVERTING STOMA	Whether the patient has a diverting stoma	No:0 Yes:1

Abbreviations: CAA: coloanal anastomosis; CAAN: colon anal anastomosis; LAR: low anterior resection; HAR: high anterior resection; ULAR: ultralow anterior resection; TNM: tumour node metastasis.

#### 2.4.2 Data pre‐processing

R software and related packages were used for data pre‐processing (R version 4.0.3 [2020‐10‐10]).

##### Missing value processing

Missing values of variables increase the difficulty of the data analysis process and bias the final results. In this study, after missing value analysis, classification variables that could not be evaluated were deleted. For continuous variables such as tumour distance to the dentate line, missing interpolation was performed using the multivariate imputation by chained equations package (MICE) in R based on the random forest method.^23^ The basic idea of MICE is that for a variable with missing values, the data of other variables are fitted to this variable and the fitted predicted values are used to fill in the missing values of this variable. In the MICE procedure, a series of regression models are run whereby each variable with missing data is modelled conditional upon the other variables in the data.

##### Data standardisation

Data standardisation can improve the convergence speed and model accuracy during data analysis. In this study, the *Z*‐Score method was used to standardise continuous variables, so that different features had the same scale, reducing the influence of the different dimensions of predictive variables on the model. To improve the accuracy of the model, the one‐hot method was used to encode discrete classification variables and detect the influence of the different variable attributes on the outcome.

##### Feature selection

Including excessive potential risk factors could lead to inconvenient model usage; thus, weakly correlated variables were removed. In this study, the Boruta algorithm was used for feature selection of included variables; all features correlated with dependent variables were screened out. The importance of variables was sorted by repeated iteration based on various categories.^24^ Finally, variables were selected based on the variable importance score, combined with clinical experience for model prediction.

##### Multicollinearity judgement

There could be a clear correlation between some prediction variables in the prediction model, which could harm the model. To avoid severe multicollinearity on the results, we conducted multicollinearity screening of independent variables by calculating the variance inflation factor (VIF) after feature selection. The formula for VIF is VIF = 1/(1 − *R* ^2^). *R* ^2^ is the correlation coefficient of the linear regression model. Thus, the greater the linear correlation accompanying the study factor, the greater the R^2^, causing an increase in VIF. In previous studies, VIF = 5 or VIF = 10, are commonly used to determine if the collinearity is strong enough to require remedial measures.^25^, ^26^ In this study, VIF > 10 was identified as having multicollinearity between variables.^27^

#### 2.4.3 Modelling methods and evaluation

In this study, five algorithms—logistic regression, support vector machine, decision tree, random forest and artificial neural network—were used to construct prediction models of LARS occurrence after colorectal cancer surgery using the research dataset. Logistic regression is a data analysis tool for predicting the outcome of classified dependent variables from a set of predictive or independent variables.^28^ Support vector machine is a classification model used as a linear or a nonlinear classifier with the largest spacing defined in the feature space. It identifies subtle differences in complex data by solving geometric hyperplanes that correctly divide training data sets.^29^ The decision tree is also a typical classification method using inductive algorithms to generate readable rules and decision trees before analysing new data to predict individual outcomes. The random forest is a set of rules for classifying data to determine the most effective way to divide it.^30^ This method involves creating and combining multiple decision trees based on the complexity of the problem. A majority vote of multiple decision trees is taken to determine a classification or prediction value.^31^ An artificial neural network is a complex network system composed of a large number of neurons with simple functions and forms connected. The artificial neural network has nonlinear mapping abilities to achieve a variety of simple or complex classifications so that the network has high fault tolerance and robustness.^32^

The caret package in R was used to construct the model, and the different built‐in functions were used to train and evaluate the five prediction models.^33^ The dataset was randomly divided into a 7:3 training set and validation set, and the models were developed using the training set. Considering the small data sample size, a 10‐fold cross‐validation method was used to test the validity of the machine‐learning models.^34^ 10‐fold cross‐validation is a resampling method in which the dataset is randomly divided into 10 pieces to improve data utilisation; one piece is used as a validation set and the remaining as training sets, with the process being repeated. Based on the average value of 10 cross‐validation results, the grid search or traversal methods were adopted for different algorithms to select the best parameters for model optimisation. Finally, the optimised models were used to test the validation set. The accuracy, specificity, sensitivity, positive predictive value, negative predictive value and other indicators of the models were obtained. Thus, the difference between the predicted and actual results could be detected to provide a basis for model selection. The receiver operating characteristics (ROC) curve and area under the curve (AUC) were used to evaluate the model's ability to predict the occurrence of LARS after colorectal cancer surgery.^29^ The Brier score, a metric in statistics to measure the accuracy of probabilistic forecasts, was used to evaluate the calibration degree of the models. The value of the Brier score is always between 0 and 1; a model with perfect skill has a score of 0, and the poorest model has a score of 1.^35^

**FIGURE 1:**
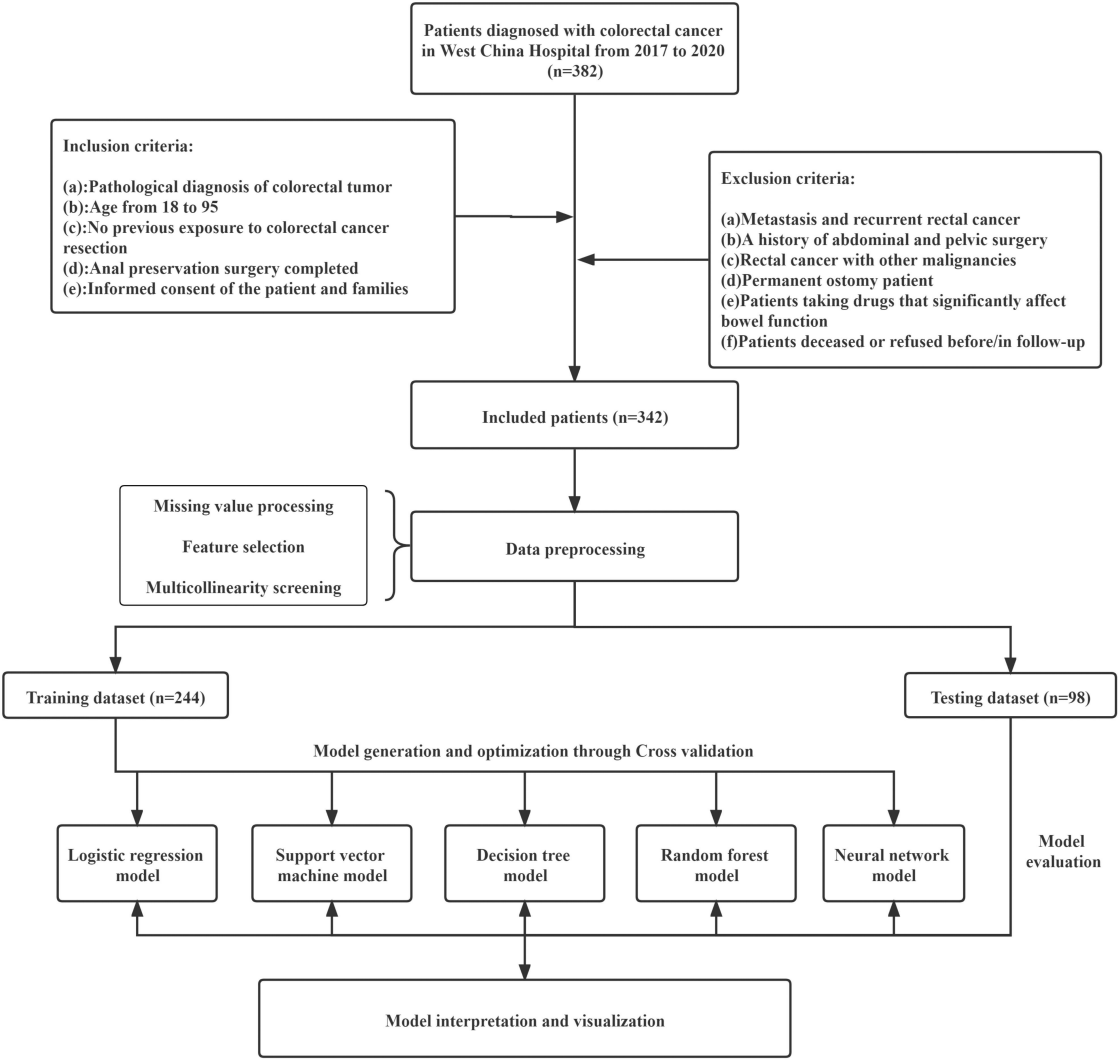
The flow gram of the study.

Finally, the results of the model are explained visually. For the logistic regression model, a nomogram was constructed to integrate the results of multiple predictive indicators and express the relationship between each variable. The results of the decision tree model are displayed via lattice packages in R, which reflects the classification nodes between the predicted variables and induces the occurrence probability of LARS from different decision paths.^36^ The results of the support vector machine, random forest and artificial neural network models are difficult to visualise due to the complexity of their algorithms. This study attempts to explain the importance of predictive variables by using the Shapley value for the above three models. The Shapley value can display the contribution of each predictive variable to the outcome variable and the positive and negative relationship between each variable and the target while considering the interaction between different variables.^37^ The Shapley value method was first proposed by Shapley L.S. to estimate the importance of an individual player in a collaborative team.^38^ In machine learning, the participants are the features you input, and the collective expenditures are the model predicted outcomes. Shapley values directly assigns contribution values to variables according to their marginal contribution to the model, thus ignoring the specific process of contribution assignment.^39^ The detailed research process of this study is shown in Figure 1.

### 2.5 Statistical analysis

Statistical analysis was performed using R (R Version 4.0.3 [2020‐10‐10]).^40^ Patients were divided into a LARS group and a No LARS group based on the follow‐up results. We compared the characteristics of baseline information, including demographic and clinicopathological data of both the groups. The Kolmogorov–Smirnov test was used to evaluate the normality of the quantitative data. The quantitative variables were represented by mean and standard deviation (SD) when they were normally distributed, and by the median and interquartile range (IQR) when they were not. The Student's *t*‐test was used for inter‐group comparison of normally distributed quantitative variables, and the Kruskal–Wallis rank‐sum test was used for comparison of non‐normally distributed quantitative variables. The classification data were expressed by frequency and percentage, and the chi‐squared (*χ* ^2^) test was used for inter‐group comparison.

## 3 RESULT

### 3.1 Characteristic

The patients' clinical baseline information, training set and validation set are summarised in Table 2. A total of 342 patients with colorectal cancer were included in this study, with the results showing LARS incidence of 48.5% (166/342). The average ages of the LARS and No LARS groups were 59.08 and 58.66 years old, respectively, with no statistical significance (*p* = 0.844). Patients with LARS had a higher proportion of neoadjuvant therapy and a diverting stoma before surgery, with the tumour being closer to the dentate line. After colorectal cancer surgery, in the LARS group, patients had earlier first postoperative exsufflation and a higher risk of leakage and reproductive dysfunction. Patients in the LARS and the No LARS groups also showed statistical differences in the tumour node metastasis (TNM) stage, obstruction, surgical type, direction and tumour location.

**TABLE 2:** Characteristic of patients between LARS group and non‐LARS group.

	LARS (*n* = 166)	No LARS (*n* = 176)	*p*	training dataset (*n* = 244)	Validation dataset (*n* = 98)	*p*
Age	59.08 ± 11.02	58.66 ± 11.50	0.844	59.38 ± 11.47	58.80 ± 10.83	0.654
Sex			0.733			0.230
Male	103 (62.0)	105 (60.3)		143 (58.6)	65 (66.3)	
Female	63 (38.0)	71 (39.7)		101 (41.4)	33 (33.7)	
BMI	23.51 ± 3.05	23.15 ± 3.07	0.215	23.37 ± 3.01	23.23 ± 3.19	0.713
Degree			0.518			0.899
Primary school	54 (32.5)	59 (33.5)		81 (33.2)	32 (32.7)	
Junior high school	44 (26.5)	45 (25.6)		62 (25.4)	27 (27.6)	
High school	28 (16.9)	21 (11.9)		37 (15.2)	12 (12.2)	
University degree or above	40 (24.1)	51 (29.0)		64 (26.2)	27 (27.5)	
Career			0.313			0.907
Farmer	57 (34.3)	49 (27.8)		73 (29.9)	33 (33.7)	
Worker	21 (12.7)	27 (15.3)		55 (22.5)	21 (21.4)	
Unemployed or retired	48 (28.9)	64 (36.4)		82 (33.6)	30 (30.6)	
Other	40 (24.1)	36 (20.5)		34 (13.9)	14 (14.3)	
Diabetes			0.789			1.000
Yes	34 (20.5)	33 (18.8)		48 (19.7)	19 (19.4)	
No	132 (79.5)	143 (81.2)		196 (80.3)	79 (80.6)	
Hypertension			0.625			0.743
Yes	58 (34.9)	67 (38.1)		91 (37.3)	34 (34.7)	
No	108 (65.1)	109 (61.9)		153 (62.7)	64 (65.3)	
Reproductive dysfunction			0.0365*			0.930
Yes	98 (59.0)	83 (47.2)		130 (53.3)	51 (52.0)	
No	68 (41.0)	93 (52.8)		114 (46.7)	47 (48.0)	
Neoadjuvant therapy			<0.001***			0.124
Yes	65 (39.2)	35 (19.9)		65 (26.6)	35 (35.7)	
No	101 (60.80	141 (80.1)		179 (73.4)	63 (64.3)	
Chemotherapy			1.000			0.422
Yes	88 (53.0)	94 (53.4)		126 (51.6)	56 (57.1)	
No	78 (47.0)	82 (46.6)		118 (48.4)	42 (42.9)	
Obstruction			0.009**			0.321
Severe	22 (13.3)	44 (25.0)		49 (20.1)	17 (17.3)	
Light	53 (31.9)	59 (33.5)		74 (30.3)	38 (38.8)	
No	91 (54.8)	73 (41.5)		121 (49.6)	17 (17.3)	
TNM			0.014*			0.157
I	65 (39.2)	41 (23.3)		74 (30.3)	32 (32.7)	
II	46 (27.7)	60 (34.1)		70 (28.7)	36 (36.7)	
III	48 (28.9)	62 (35.2)		87 (35.7)	23 (23.5)	
IV	7 (4.2)	13 (7.4)		13 (5.3)	7 (7.1)	
Location			<0.001***			0.649
Lower rectum	116 (69.9)	42 (23.9)		109 (44.7)	49 (50.0)	
Middle rectum	12 (7.2)	68 (38.6)		57 (23.4)	23 (23.5)	
Upper rectum	11 (6.6)	46 (26.1)		41 (16.8)	16 (16.3)	
Colon	27 (16.3)	20 (11.4)		37 (15.2)	10 (10.2)	
Size	1.89 (0.77, 3.77)	1.58 (0.57, 3.11)	0.051	2.02 (0.70, 3.54)	1.50 (0.62, 3.15)	0.218
Direction			0.011*			0.912
Annular	50 (30.1)	80 (45.5)		91 (37.3)	39 (39.8)	
Anterior	80 (48.2)	63 (35.8)		105 (43.0)	38 (38.8)	
Lateral	11 (6.6)	5 (2.8)		11 (4.5)	5 (5.1)	
Posterior	25 (15.1)	28 (15.9)		37 (15.2)	16 (16.3)	
Surgical type			<0.001***			0.986
CAA	27 (16.3)	17 (9.7)		29 (11.9)	15 (15.3)	
CAAN	62 (37.3)	22 (12.5)		54 (22.1)	30 (30.6)	
HAR	13 (7.8)	66 (37.5)		54 (22.1)	25 (25.5)	
LAR	26 (15.7)	54 (30.7)		59 (24.2)	21 (21.4)	
ULAR	38 (22.9)	17 (9.7)		48 (19.7)	7 (7.1)	
Anastomotic type			0.120			0.528
End‐to‐end	142 (85.5)	161 (91.4)		214 (87.7)	89 (90.8)	
End‐to‐side	24 (14.5)	15 (8.6)		30 (12.3)	9 (9.2)	
Contracted pelvis			0.253			0.187
Severe	22 (13.3)	14 (8.0)		29 (11.9)	7 (7.1)	
Light	89 (53.6)	96 (54.5)		135 (55.3)	50 (51.0)	
No	55 (33.1)	66 (37.5)		80 (32.8)	41 (41.9)	
Adhesions of pelvic organs			0.510			0.443
Severe	5 (3.0)	9 (5.1)		10 (4.1)	4 (4.1)	
Light	71 (42.8)	68 (38.6)		94 (38.5)	45 (45.9)	
No	90 (54.2)	99 (56.3)		140 (57.4)	49 (50.0)	
Surgical comorbidity			0.079			0.769
Yes	67 (40.4)	54 (30.7)		88 (36.1)	33 (33.7)	
No	99 (59.6)	122 (69.3)		156 (63.9)	65 (66.3)	
Exsufflation	2.00 (1.00, 2.00)	2.00 (2.00, 3.00)	<0.001***	2.00 (1.00, 3.00)	2.00 (1.00,3.00)	0.468
Risk of leakage			0.037*			0.228
High	48 (28.9)	33 (18.8)		53 (21.7)	28 (28.6)	
Low	118 (71.1)	143 (81.3)		191 (78.3)	70 (71.4)	
Diverting stoma			<0.001***			0.403
Yes	122 (73.5)	56 (31.8)		123 (50.4)	53 (54.0)	
No	44 (26.5)	120 (68.2)		121 (49.6)	43 (43.9)	

Abbreviations: CAA, colonanal anastomosis; CAAN, colon anal anastomosis; HAR, high anterior resection; LAR, low anterior resection; LARS, low anterior resection syndrome; TNM, tumour node metastasis; ULAR, ultralow anterior resection.

*p ≤ 0.05, **p ≤ 0.01, ***p ≤ 0.001.

These differences were all statistically significant (*p* < 0.05). There was also no clear difference between the data of the training set and the verification set by random grouping with balanced data distribution.

### 3.2 Feature selection

**FIGURE 2:**
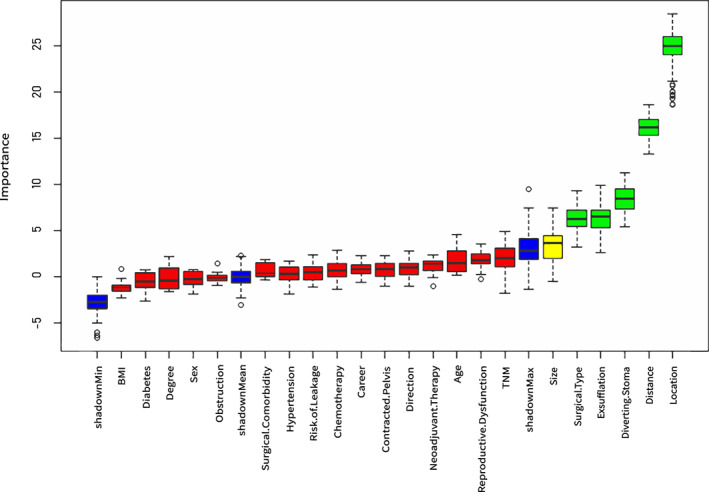
The ranking of the importance of all variables. Different colours represent different importance. Green means the variable is important to the model, yellow means generally important and red means not important. Blue is a random variable automatically generated by the algorithm and is not included in the analysis.

In this study, the average missing rate of the overall data was 5.04%, and the missing data were interpolated by using MICE package in R. The specific information about the missing values can be seen in additional file 1. The Boruta Package in R was used to rank the importance of the included variables.^24^ The results showed that ‘location, distance, surgical type, diverting stoma and exsufflation’ had the most influence on LARS (Figure 2). Moreover, based on clinical experience and the convenience of model application, ‘age’ was included in the analysis after discussion. Multicollinearity judgement was conducted on the variables after feature selection with results showing that the variance inflation factor values of all variables were less than 10, and thus indicating no serious collinearity problem. Finally, location, distance, surgical type, diverting stoma, exsufflation and age were included as indicators in the model construction.

### 3.3 Performance of models

#### 3.3.1 The logistic regression model

**FIGURE 3:**
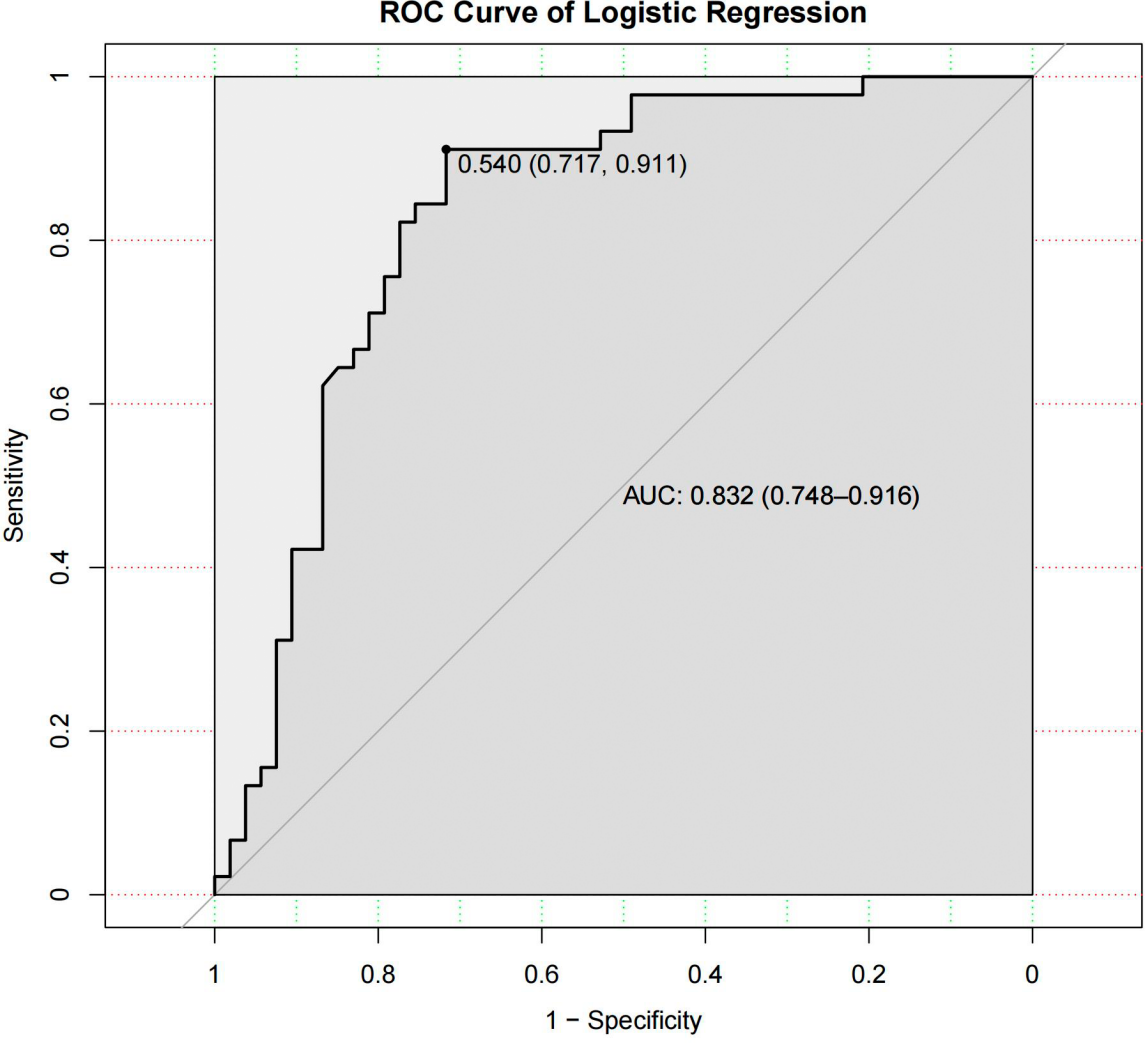
The ROC curve of logistic regression. The area under the curve (AUC) for the prediction model based on the logistic regression algorithm was 0.832 with a 95% confidence interval of (0.748, 0.916).

**FIGURE 4:**
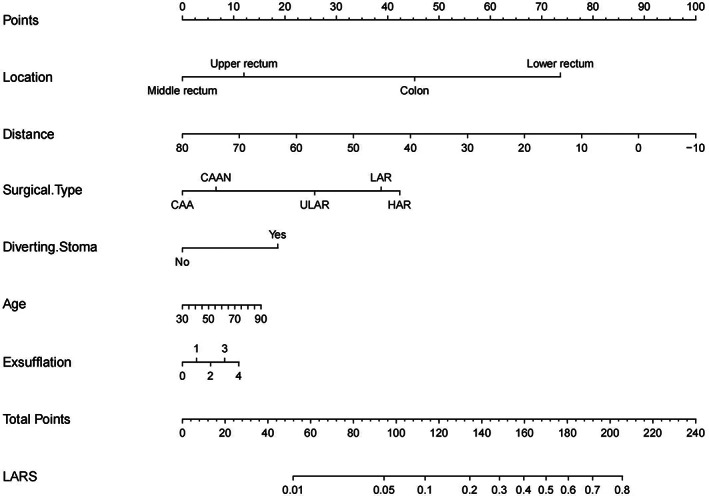
The nomogram of the logistic regression model. The characteristics of patients in terms of tumour location, age, etc. can correspond to different points in the first column. The scores of these different aspects are added together to obtain a total points, which corresponds to the probability of occurrence of ‘LARS’ in this last column. For example, if a patient is evaluated with a total points of 160, the probability of having LARS is approximately 40% referred to the nomogram.

The logistic regression model was constructed by using the variables after feature selection and multicollinearity screening. The results of the logistic regression model are summarised in Table 3. Age, distance and exsufflation exhibit negligible risk for LARS and are not statistically significant. Low rectal cancer is a significant risk factor, and the odds ratio (OR) is 3.01, but the results are not statistically significant (*p* > 0.05). Middle rectal cancer is a protective factor (OR = 0.17), with the difference being statistically significant (*p* = 0.005). Different types of surgery have different risk levels for LARS, but these differences are not statistically significant. This may be due to the many types of surgical methods, resulting in a small sample size of each surgical type, reducing the effectiveness of the statistical test. The prediction accuracy of the prediction model was 80.6%, with a 95% confidence interval (CI) (0.714, 0.879). The ROC curve was produced based on the model results, which showed that the AUC was 0.832, the optimal threshold was 0.540, the corresponding sensitivity was 0.911, and the specificity was 0.717. The model exhibited good predictive ability (Figure 3). The Brier score of the model was 0.159, indicating that the prediction results of the model were in good agreement with the actual outcome. To improve the convenience of using the model, a nomogram was made based on the results (Figure 4). The corresponding line segment on the nomogram is obtained through patient information, and then the corresponding score is obtained by comparing item ‘Points’. Then, the scores obtained by patients in different projects are added up, and the probability of LARS can be obtained by comparing item ‘Total Points’ and item ‘LARS’.

**TABLE 3:** Results of logistic regression model.

Predictors	Odds ratios	95% CI	*p*
Age	1.01	0.98–1.01	0.492
Distance	0.96	0.84–1.04	0.455
Exsufflation	1.11	0.74–1.69	0.610
Diverting Stoma	2.06	0.72–5.88	0.174
Location [Colon]	–	–	Reference
Location [Lower rectum]	3.01	0.99–9.87	0.057
Location [Middle rectum]	0.17	0.05–0.56	0.005
Location [Upper rectum]	0.27	0.05–1.41	0.116
Surgical Type [CAA]	–	–	Reference
Surgical Type [CAAN]	1.29	0.44–3.71	0.638
Surgical Type [HAR]	5.18	0.66–45.34	0.125
Surgical Type [LAR]	4.51	0.87–26.24	0.081
Surgical Type [ULAR]	2.72	0.85–9.37	0.099

Abbreviations: CAA, coloanal anastomosis; CAAN, colon anal anastomosis; HAR, high anterior resection; LAR, low anterior resection; TNM, tumour node metastasis; ULAR, ultralow anterior resection.

#### 3.3.2 The support vector machine model

**FIGURE 5:**
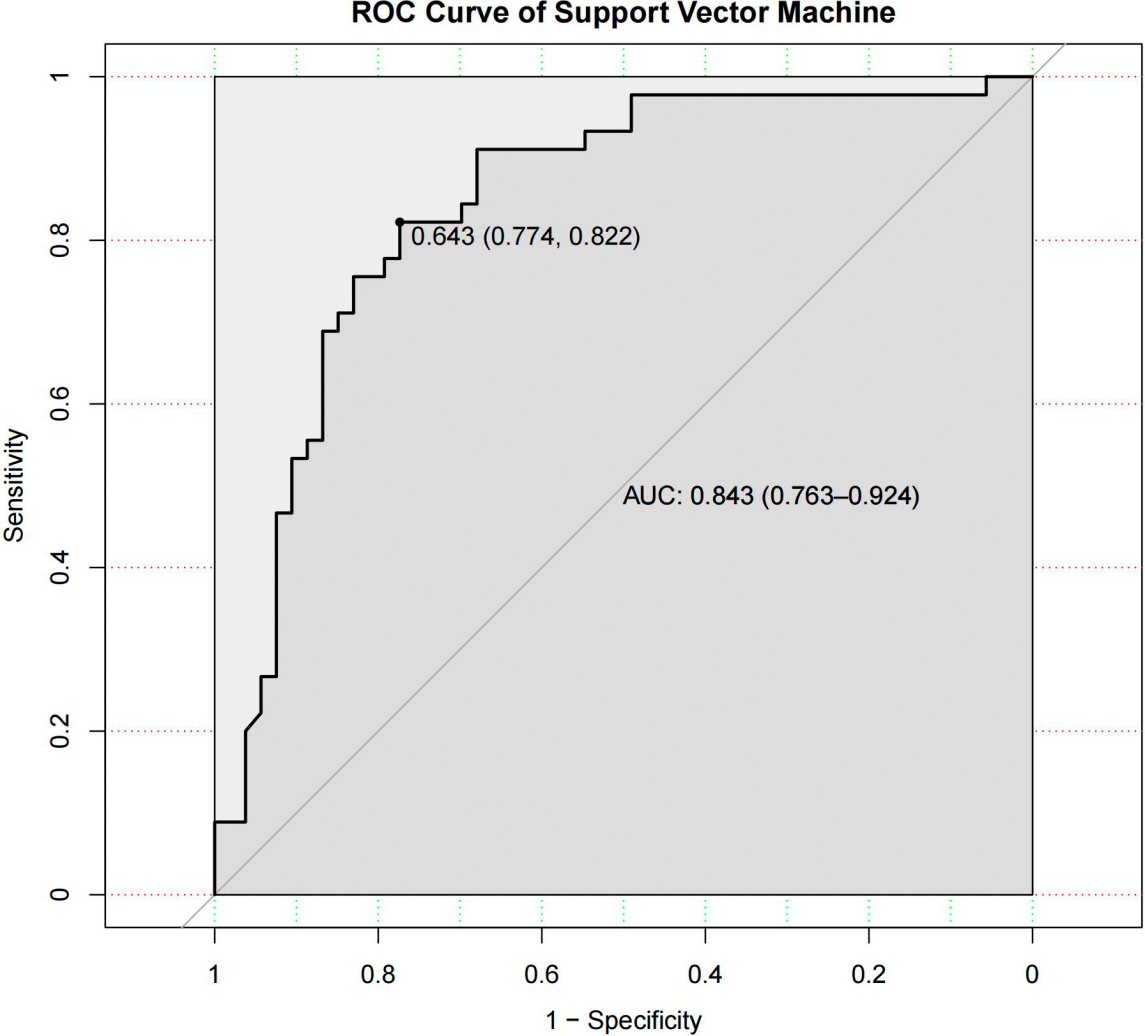
The ROC curve of support vector machine. The area under the curve (AUC) for the prediction model based on the support vector machine algorithm was 0.843 with a 95% confidence interval of (0.763, 0.924).

**FIGURE 6:**
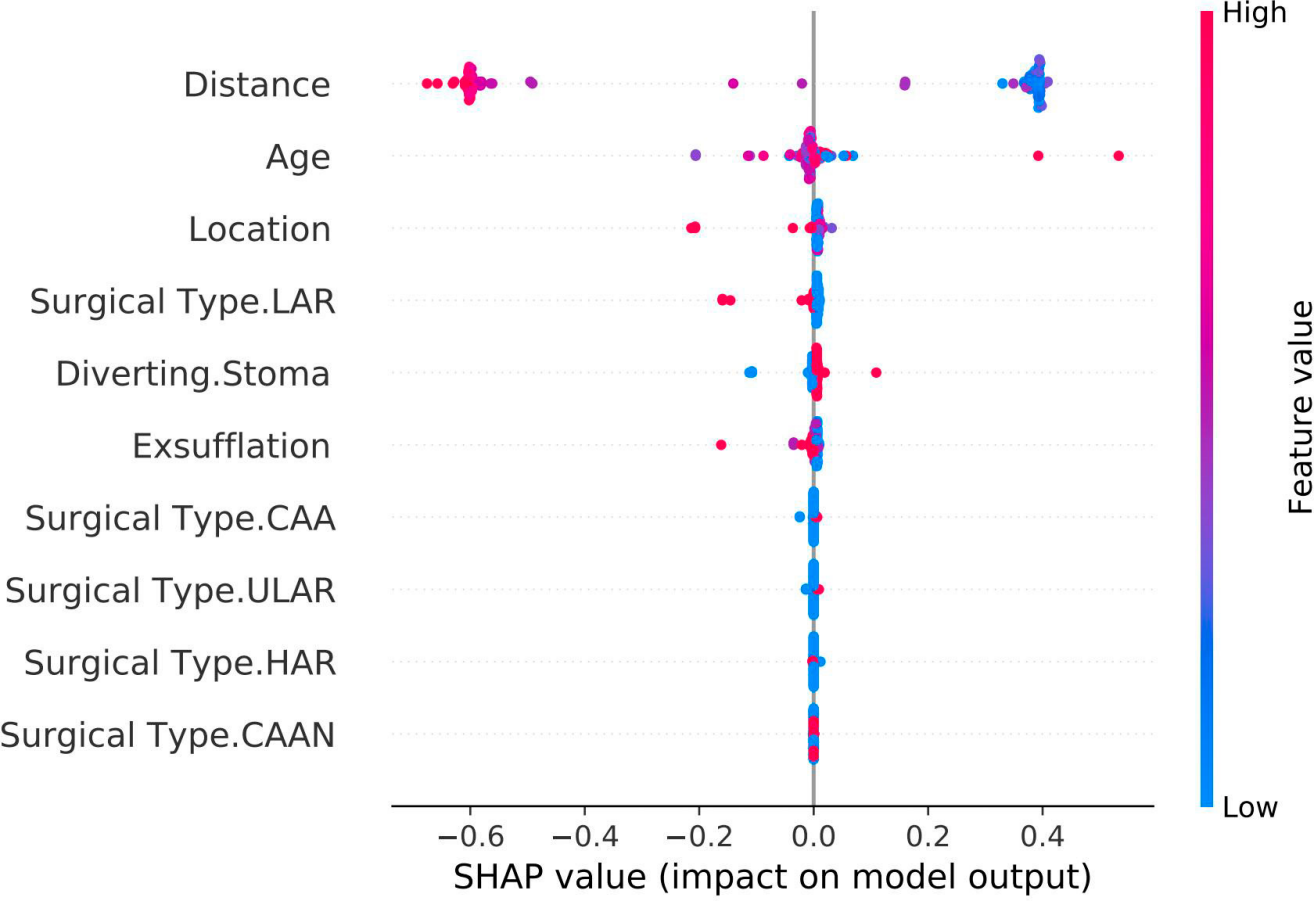
The interpretation of support vector machine model by Shapley value. The importance of different variables decreases from top to bottom. For a single variable such as ‘Distance’, the colour of the dot represents the value of the variable, the closer to red the higher the corresponding value, that is the larger the ‘Distance’ value. The distributions of the dots represent the degree of influence on the model, that is, the likelihood of LARS occurring. The diagram shows that the smaller the tumour distance, the higher the likelihood of LARS occurrence in patients.

The caret package in R was used to conduct support vector machine modelling for the variables included in the feature selection. As the support vector machine algorithm adopts different combinations of variables to form support vectors and uses them to predict the outcome, it is difficult to intuitively present the results. The ROC curve was produced based on the results, which showed that the AUC was 0.843, and the accuracy was 79.6%. The sensitivity and specificity corresponding to the optimal threshold value were 0.822 and 0.774, respectively (Figure 5). The Brier score of the model was 0.168, indicating that the prediction results of the model were in good agreement with the actual outcome. The Shapley value was used for interpretation, and an abstract figure was subsequently generated (Figure 6). The results showed that, in this model, the three most important predictive variables were distance, age and location. For instance, considering the predictive variable ‘distance’ (the most influential factor), the lower the distance patients have, the higher the Shapley value they contributed. This demonstrated that the lower the distance from the patient's tumour to the dentate line, the higher the risk of LARS.

#### 3.3.3 The decision tree model

**FIGURE 7:**
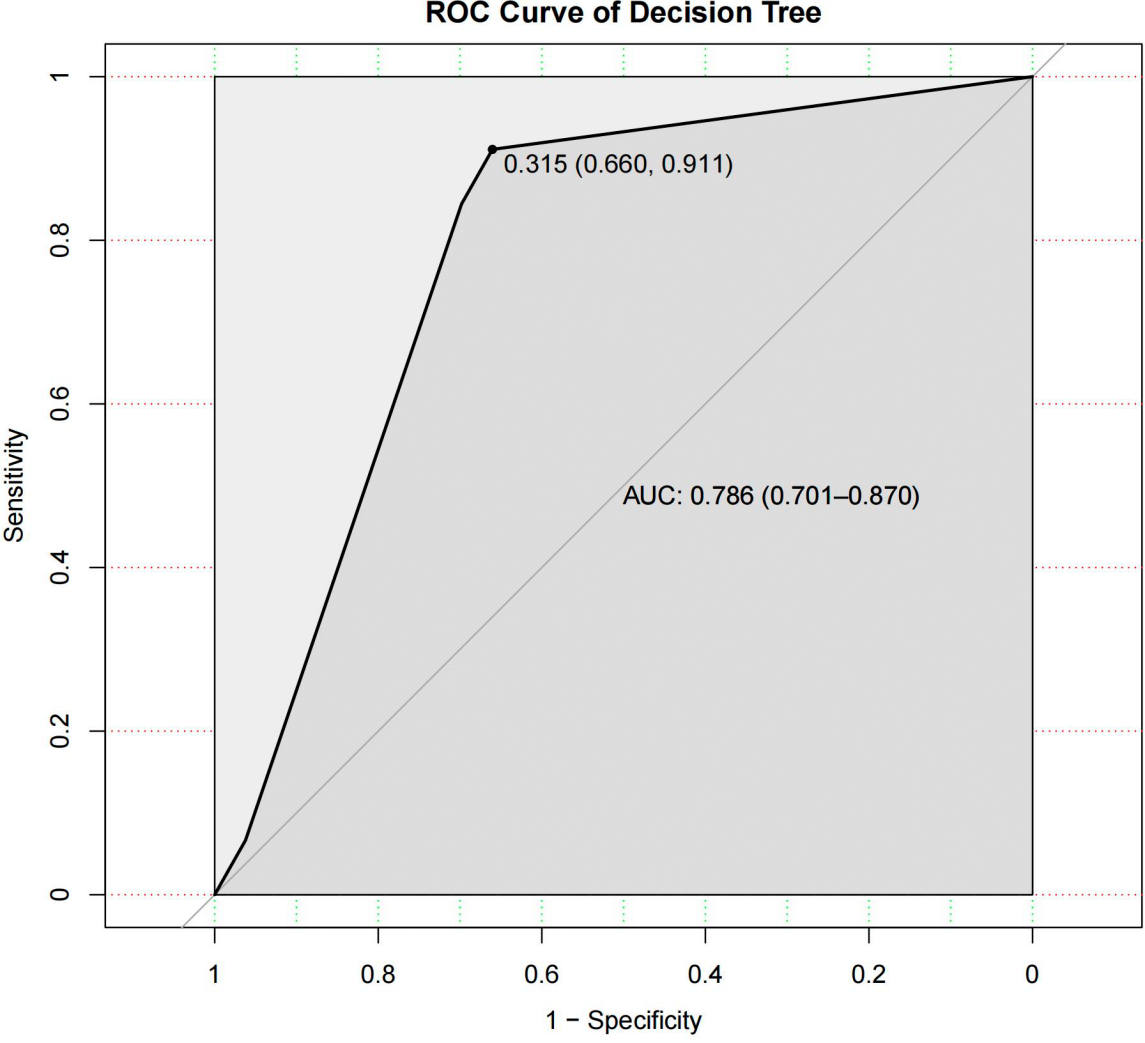
The ROC curve of decision tree. The area under the curve (AUC) for the prediction model based on the decision tree algorithm was 0.786 with a 95% confidence interval of (0.701, 0.870).

**FIGURE 8:**
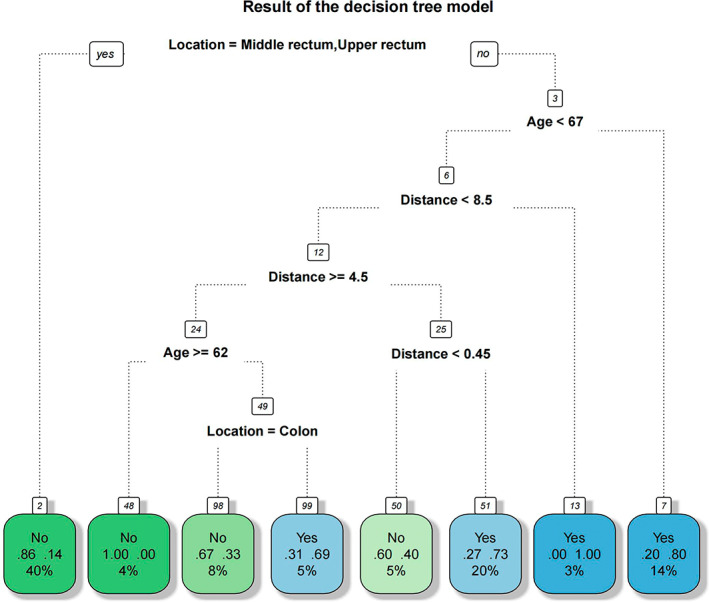
The visualisation results of the decision tree model. Patients were progressively screened according to different aspects of their characteristics from top to bottom by the conditions shown in the figure. And then the final probabilities corresponding to the occurrence of LARS in patients were obtained. A darker blue colour means that the patient has a higher probability of developing LARS, while a darker green colour means the opposite.

The rpart package in R is used for model development. The results showed ‘location’ as the most effective indicator of LARS occurrence, followed by age and the distance between the tumour and the dentate line. With decreasing distance and increasing age, the probability of LARS occurrence increases. Similar results can be seen in Figure 4. The prediction accuracy of the model was 77.6%, with a 95% CI (0.680, 0.854). The ROC curve was established based on the results of the decision tree model. The results show the AUC was 0.786, and the sensitivity and specificity under optimal threshold were 0.660 and 0.911, respectively (Figure 7). In terms of calibration, the Brier score of the model was 0.172. And the visualisation results of the decision tree model can be seen in Figure 8.

#### 3.3.4 The random forest model

**FIGURE 9:**
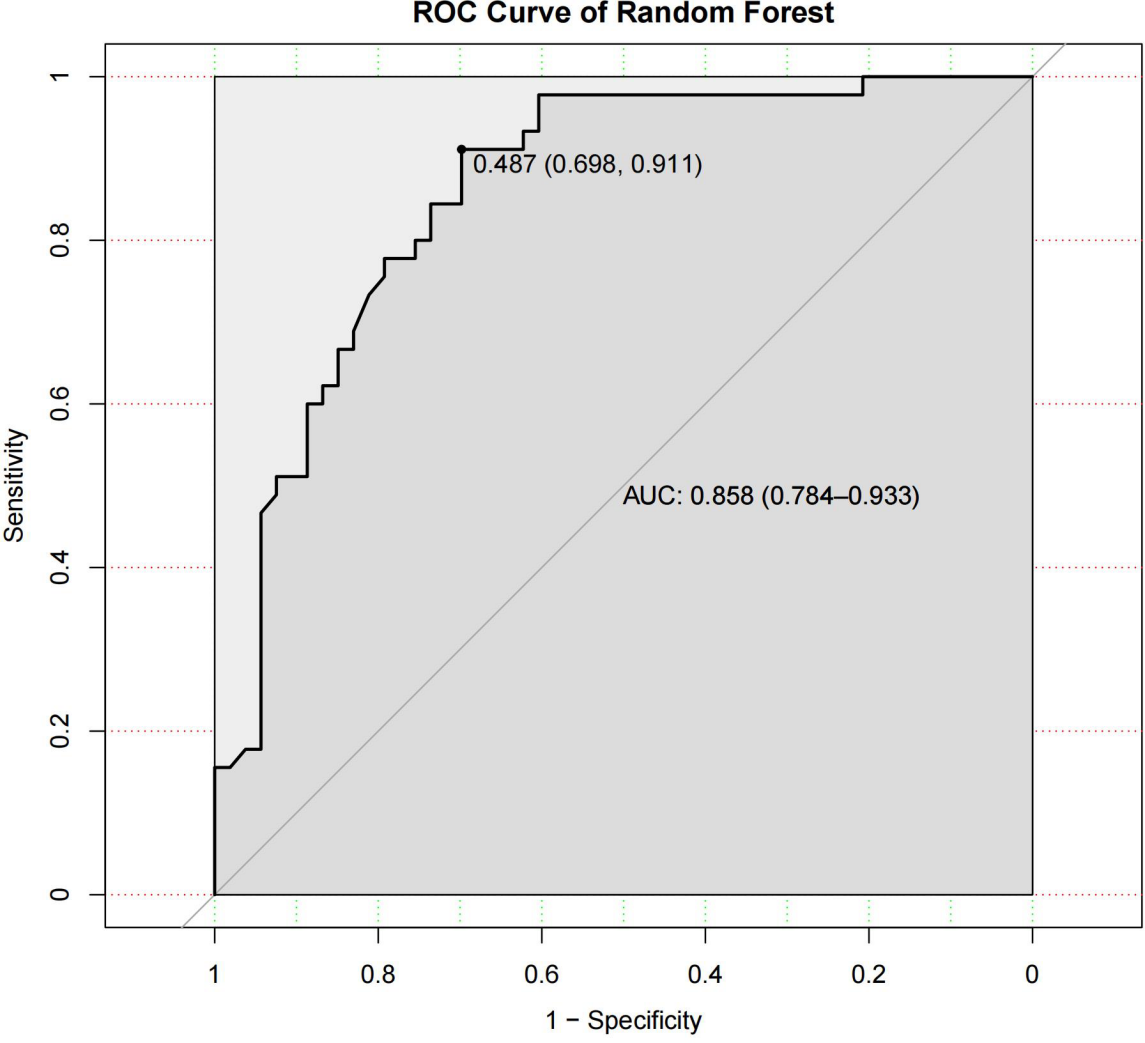
The ROC curve of random forest. The area under the curve (AUC) for the prediction model based on the random forest algorithm was 0.858 with a 95% confidence interval of (0.784, 0.933).

**FIGURE 10:**
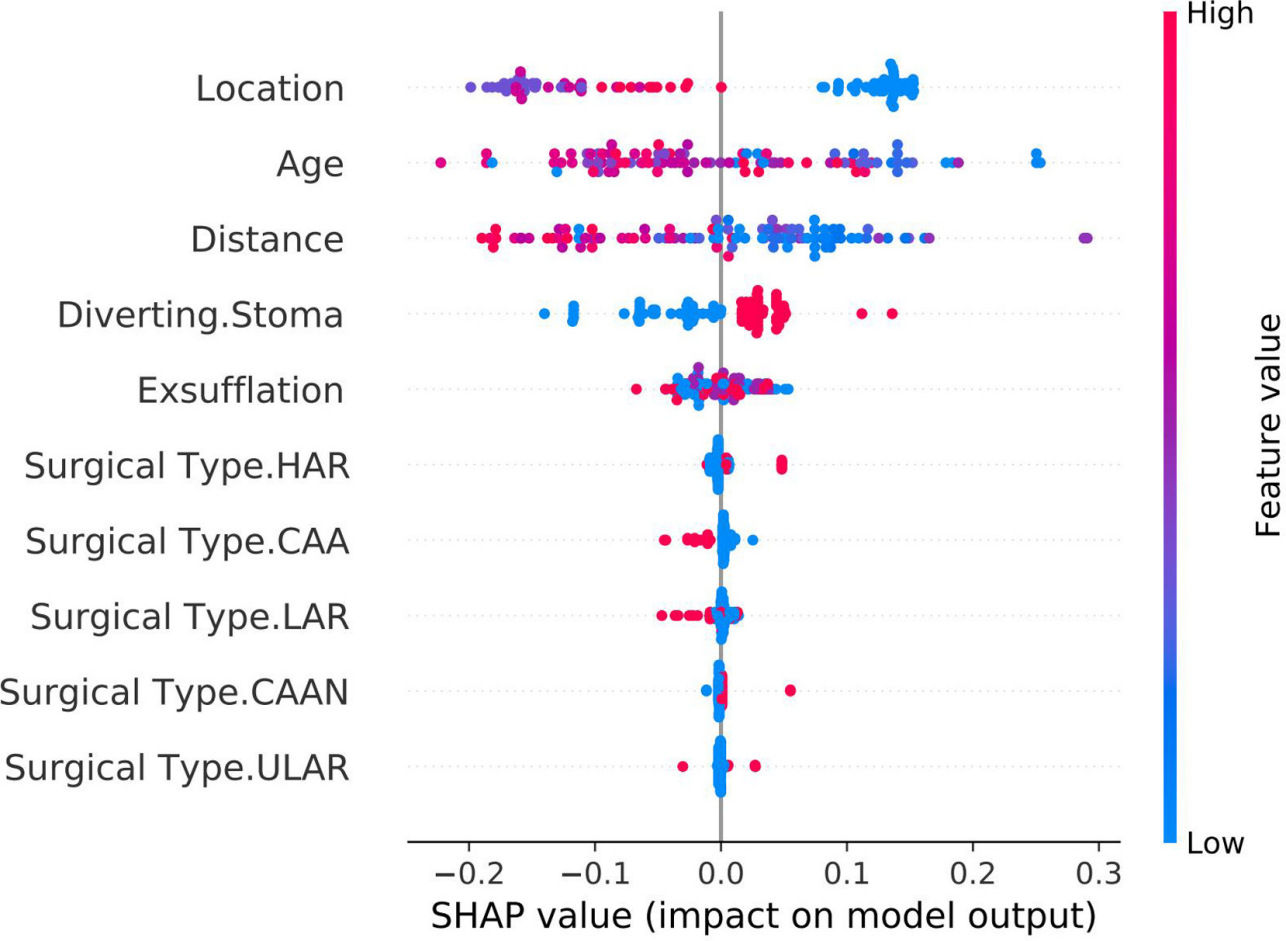
The interpretation of random forest model by Shapley value. The importance of the variables decreases from top to bottom. The colour of the dots represents the value of the variable itself, with red meaning larger values and blue representing smaller values. The distribution of the dots represents the degree of influence on the occurrence of LARS, with the closer to the right the higher the degree of influence. The variables that have a significant impact on the model are ‘Location’, ‘Age’ and ‘Distance’ in order.

The random forest algorithm is an integrated learning algorithm, which is a set of decision trees. The ROC curve was produced based on the results, which showed the prediction accuracy of the model to be 79.6%, with a 95% CI (0.703, 0.871). The AUC was 0.858, and the sensitivity and specificity under the optimal threshold were 0.911 and 0.698, respectively (Figure 9). The Brier score of the model was 0.165. The Shapley value was used to explain the result of the model, as shown in Figure 10. The three predictive variables with the most influence on the model were ‘location, age, distance’, not dissimilar to the results of the support vector machine model. In the explanatory graph of Shapely values, the colour of the data points represents the high or low value of the different variables, while the position in the graph represents the value of the contribution to the model. The higher the Shapely value corresponding to a data point, the higher the contribution to the occurrence of the outcome indicator. As can be seen in the graph, most of the tumours in lower locations contribute high Shapely values to the model, for example, meaning that the lower the location of the tumour, the higher the incidence of LARS.

#### 3.3.5 The artificial neural network model

**FIGURE 11:**
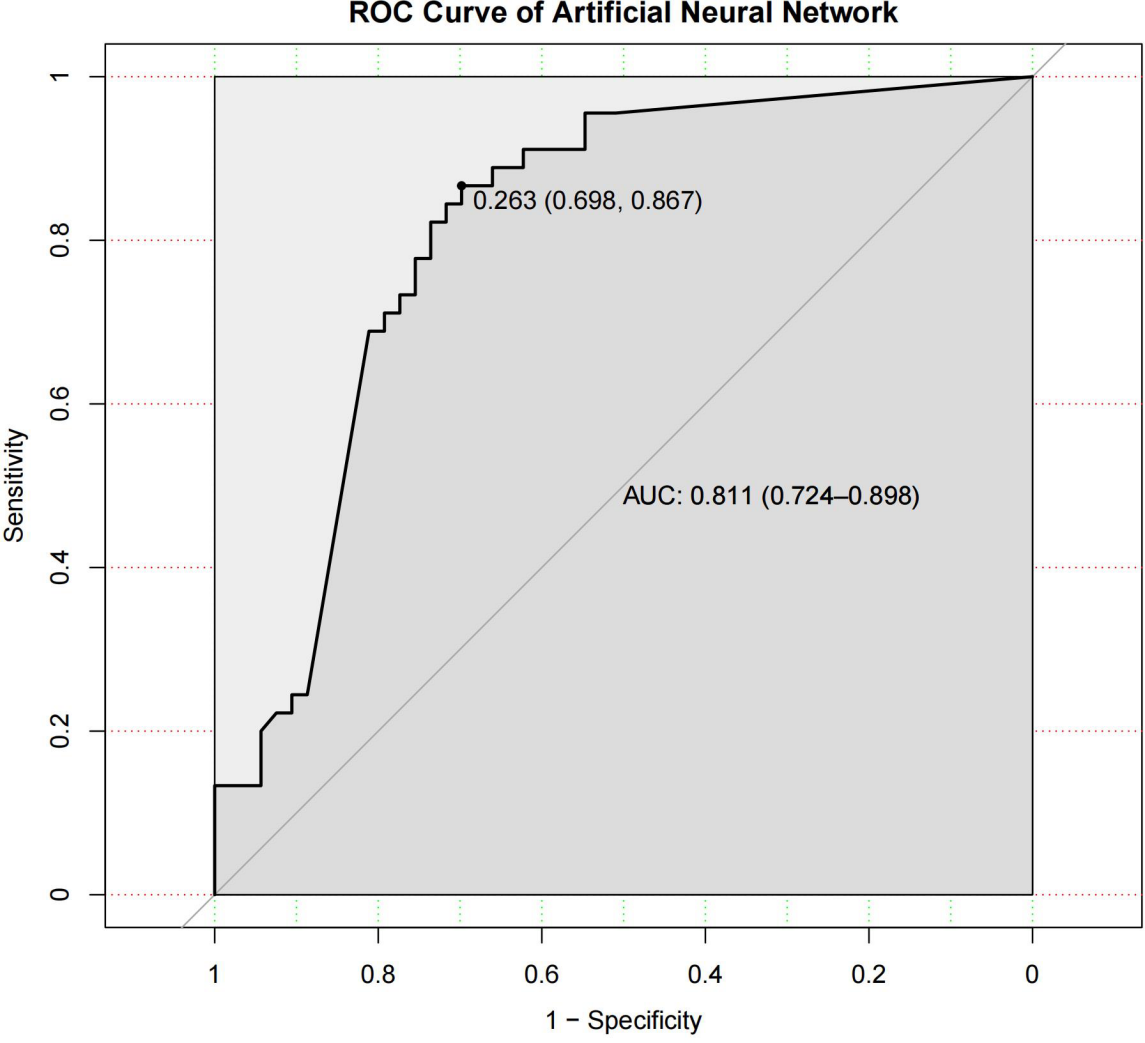
The ROC curve of artificial neural network. The area under the curve (AUC) for the prediction model based on the artificial neural network algorithm was 0.811 with a 95% confidence interval of (0.724, 0.898).

**FIGURE 12:**
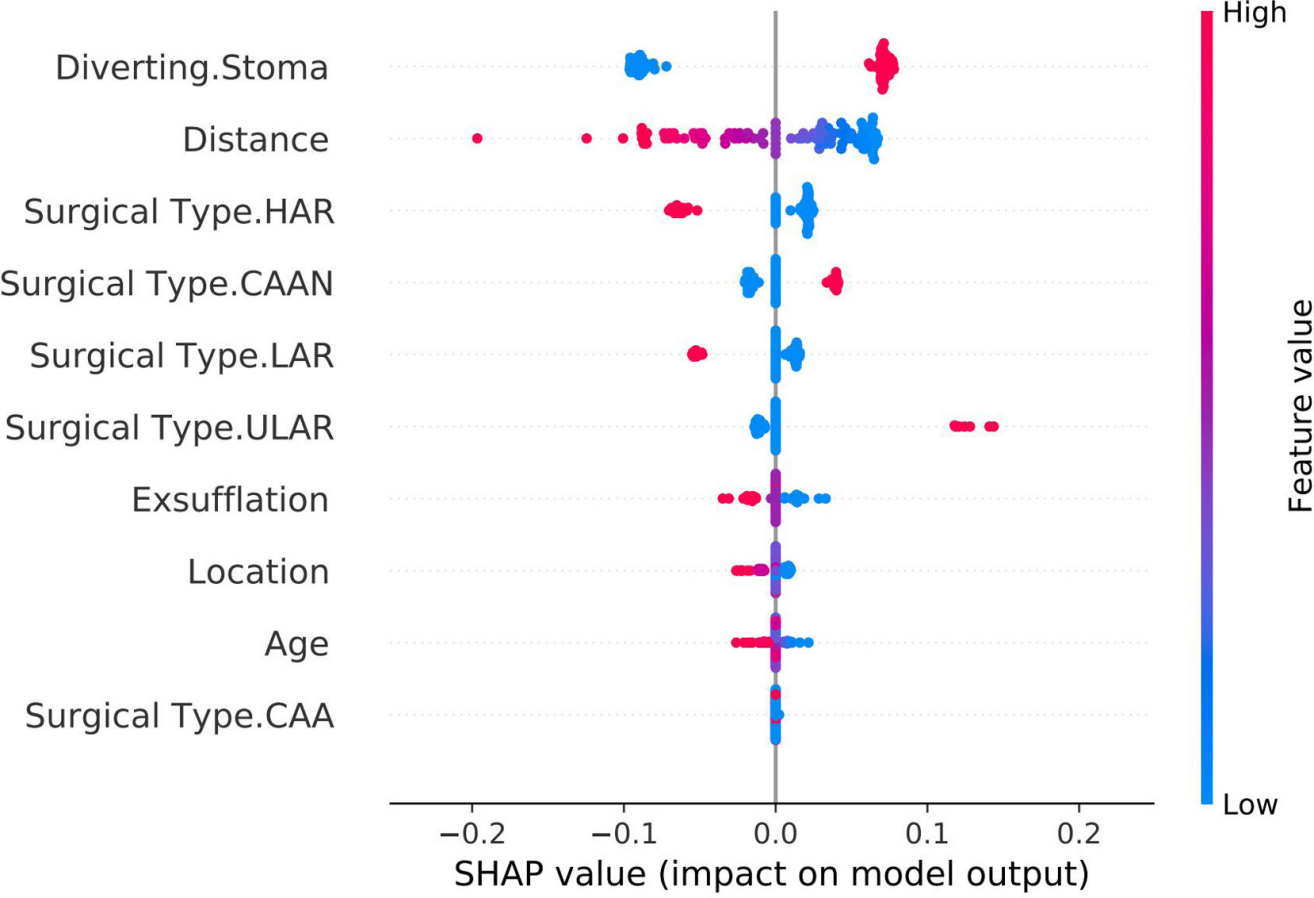
The interpretation of artificial neural network model by Shapley value. The variables that have a significant impact on the model are ‘Diverting Stoma’, ‘Distance’ and ‘Surgical Type’ in order.

The caret package in R was used to construct the model, whose reliability was evaluated based on the AUC value of the ROC curve. The results showed that the AUC was 0.811, the accuracy was 0.776 at the optimal threshold with a 95% CI (0.680, 0.854). The sensitivity and specificity of the model were 0.867 and 0.698, respectively (Figure 11). The Brier score of the model was 0.175, which was poor compared with the other models. The Shapley value was used to explain the model, and the result is shown in Figure 12. Unlike the results of the random forest and support vector machine models, the three variables with the most influence on the artificial neural network model were ‘diverting stoma, distance and surgical type’. Patients with a diverting stoma and a short distance from the tumour to the dentate line were more likely to have LARS after surgery.

### 3.4 Comparison of model performance

The results of the prediction models obtained using the five algorithms were compared based on the optimal threshold, the results being summarised in Table 4. The accuracy of the five models was over 75%, there being no obvious difference. The sensitivity of the logistic regression, decision tree and random forest models all reached 0.911. The support vector machine model had the highest specificity and positive predictive value (0.774 and 0.755, respectively). The logistic regression model had the highest negative predictive value (0.905). For the overall prediction performance of the model, the random forest model proved to be the best, with an AUC of 0.858.

**TABLE 4:** Performance comparison of five models.

	Cut‐off	Sen	Spe	PPV	NPV	Accuracy (95% CI)	AUC (95% CI)	BS
LR	0.54	0.911	0.717	0.732	0.905	0.806 (0.714, 0.879)	0.832 (0.748, 0.916)	0.159
SVM	0.648	0.822	0.774	0.755	0.837	0.796 (0.703, 0.871)	0.843 (0.762, 0.924)	0.168
DT	0.315	0.911	0.660	0.695	0.897	0.776 (0.680, 0.854)	0.786 (0.701, 0.870)	0.172
RF	0.508	0.911	0.698	0.719	0.902	0.796 (0.703, 0.871)	0.858 (0.787, 0.934)	0.165
ANN	0.263	0.867	0.698	0.709	0.886	0.776 (0.680, 0.854)	0.811 (0.724, 0.898)	0.175

Abbrevations: ANN, artificial neural network; AUC, area under curve; BS, brier score; DT, decision tree; LR, logistic regression; NPV, negative predictive value; PPV, positive predictive value; RF, random forest; SEN, sensitivity; SPE, specificity; SVM, support vector machine.

## 4 DISCUSSION

LARS often occurs after LAR for colorectal cancer patients, seriously affecting the postoperative quality of life of patients.^41^ LARS manifests in patients with varying degrees of postoperative symptoms—such as increased frequency of defecation, difficult urgent discharge, anal swelling and defecation incontinence—with a probability of 30%–70%, which is consistent with the results of this study indicating 47.4%. The LARS scale is mainly used as a tool to evaluate LARS in clinical practice, but there is a lack of a simple and easy prediction method for the occurrence of LARS in colorectal cancer patients. In this study, machine‐learning algorithms were used to develop prediction models based on the medical information of colorectal cancer patients to predict the probability of LARS occurrence after surgery.

All five machine‐learning methods demonstrated good accuracy and stability, with an AUC of 0.832 for logistic regression, 0.843 for support vector machine, 0.786 for decision tree, 0.858 for random forest and 0.811 for artificial neural network model, suggesting good predictive capabilities. These models could provide clinicians and nurses with clinical suggestions to help them formulate better nursing strategies to improve the prognosis of LARS and the postoperative quality of life of their patients. This study showed that the LARS risk factors post‐surgery included tumour location, distance between the tumour and dentate line, diverting stoma, postoperative exhaust days and surgical type.

There have been few studies of predictive models of LARS after colorectal cancer until now, and in most studies, researchers have only used logistic regression analysis for risk factors and have not performed prediction and validation. In Cornish's study, neoadjuvant radiotherapy was an independent risk factor for LARS.^42^ And in Wu et al.’s study, in addition to neoadjuvant radiotherapy, low tumour and diverting stoma was also risk factors for the occurrence of LARS.^22^ This is broadly similar to our findings, but in our study neoadjuvant radiotherapy, although a risk factor for LARS in the univariate analysis, did not reflect a higher importance in the feature selection process and thus was not included in the final analysis. This does not mean, however, that neoadjuvant radiotherapy was not a risk factor for the LARS in our study. There appears to be only one predictive modelling study of LARS. Ekkarat^13^ used logistic regression for risk factor analysis and attempted a predictive modelling analysis. The results showed that radiotherapy and low anastomosis were predictors of LARS, but the AUC of the constructed model was only 0.73. In our study, the logistic regression model had an AUC of 0.83, which may be due to our inclusion of more influential factors and feature selection.

In some other previous studies, the results were more or less the same, and the risk factors were mainly one or more radiotherapy, low tumour, low anastomotic position, diverting stoma and surgical type, which were more limited.^7^, ^11^, ^13^, ^22^, ^42^, ^43^, ^44^ However, this study conducted a systematic meta‐analysis and literature review prior to the start of the study and thus included more influential factors for analysis.

Among the risk factors shown in this study, despite statistically significant differences in neoadjuvant therapy, the TNM stage, tumour size, preoperative complications and the direction between the LARS group and the No LARS group (*p* < 0.05), their contribution to the construction of the whole model was low. Reviewing the baseline information of the LARS and No LARS groups, although there was a statistical difference in TNM stage and tumour size between the two groups, the clinical difference was not significant. The tumour volume in the LARS group was only 0.31 cm^3^ larger than that in the No LARS group on average. And the difference between the two groups in tumour stage was also not significant and thus ultimately not significant for the model. Consequently, they were not included in the analysis as predictive variables considering the need for simplicity in the clinical context. There was a deviation from our initial expectations and previous studies.^45^, ^46^ After much consideration, we surmised that this deviation may have been due to the interaction between variables in the model construction. Although these factors showed differences between patients with and without LARS in single‐factor analysis, the contribution of the comprehensive analysis of all variables in the model was low.

The performance of the five prediction models developed in this study did not show significant differences under their respective optimal thresholds. Interestingly, the logistic regression model achieved the best accuracy, contrary to original assumptions. The support vector machine and decision tree models did not show many advantages. We made the following assumptions: on the one hand, these models need not have a large amount of training data to demonstrate a good classification result; however, the relationship between variables and outcomes was more likely to be linearly conforming to the logistic regression.

In terms of the overall discriminating ability of the models, the random forest model performed best, with an AUC of 0.857, showing good predictive capability. The logistic regression, decision tree and random forest models showed the same model sensitivity (0.911) and could be used as screening tools for LARS occurrence. However, considering convenience in the clinic, the logistic regression model was the best choice as it could be applied to the nomogram. The support vector machine model had the highest specificity and could be used as a diagnostic tool.

A low tumour significantly increased the incidence of LARS, which could result from the difficulty of low rectal tumour resection and high anastomotic tension, easily leading to intestinal isospasm and stenosis at the proximal end of anastomosis.^5^, ^43^, ^47^ The lower the distance between the tumour and the anal margin, the lower the position of the postoperative anastomosis. This causes a greater degree of injury to the anal sphincter (especially the internal sphincter) and the perianal nerve, thus damaging the function of rectal defecation and faecal storage, leading to the occurrence of LARS.^22^

The present study found that although a diverting stoma could negatively affect intestinal function, the time interval from the stoma establishment to the stoma closure did not affect the impairment of intestinal function or the occurrence of LARS.^48^ Preoperative radiotherapy and chemotherapy significantly increased the risk of LARS because radiation could damage the conduction of the intramuscular nerve plexus and muscle fibrosis and thus lead to increased incontinence and decreased rectal sensation.^49^

In this study, the number of days of postoperative exhaust was included in the prognostic analysis for the first time. The outcome suggests that the number of days of postoperative schedule could be used as a predictive mark of LARS, with the number of days of postoperative exhaust being later than without LARS (*p* < 0.001). This may be because patients with more severe rectal and anal function injury have worse exhaust function than general patients, with the difference being reflected in the time of first exhaust after surgery. Moreover, some studies have suggested that neuromuscular injury during surgery in colorectal cancer patients is a risk factor for LARS. As Jimenez pointed out in his study, due to male pelvic stenosis, lower abdominal nerves are more likely to be damaged through mesenteric dissection during total mesorectal excision (TME) surgery, leading to a higher risk of LARS in men.^43^, ^50^ However, intraoperative injury can be difficult to quantify, and different study groups undergo different surgical procedures, making it more difficult to assess injuries. Consequently, this study did not include intraoperative injury in the analysis of the prediction model. However, it is worth noting that in this study, there was no significant difference in LARS occurrence using end‐to‐side anastomosis or end‐to‐end anastomosis (*p* = 0.12). And it is slightly different from the results of a previous meta‐analysis.^51^ After consideration, we thought that it was mainly due to the small sample size of patients undergoing end‐to‐side anastomosis (39/342), resulting in insufficient statistical efficiency.

Currently, the relief of symptoms regarding LARS is more important after low anterior resection. In the current studies, there is no specific treatment for LARS. In terms of management after low anterior resection, conservative therapies (e.g. pelvic floor rehabilitation or colonic irrigation) or minimally invasive therapies (e.g. sacral nerve stimulation) are the basis for current and future treatment of LARS. A multimodal approach, rather than monotherapy, may be the best management option for these patients. Up to 18% of patients who undergo anterior resection have internal anal sphincter injury and may have compromised innervation during rectal motility.^52^ Whereas postoperative neointestinal muscle motility appears to be degraded by intraoperative manipulation resulting in residual colonic denervation.^53^ Sacral nerve stimulation has been shown to improve faecal incontinence and delayed defecation in normal and sphincter‐impaired patients as well as in patients affected by LARS. The mechanism of action of sacral nerve stimulation was initially thought to be a direct effect on the anal sphincter to increase resting and squeezing pressure. Due to the overwhelming evidence of extra‐anorectal effects, it seems likely that the effects of sacral nerve stimulation on anorectal function occur at the level of pelvic afferents.^54^ In addition to sacral nerve stimulation, most studies have reported significant improvements in faecal frequency, urinary incontinence, severity of faecal incontinence and health‐related quality of life following pelvic floor muscle training and biofeedback.^55^ For example, patients were instructed to use a biofeedback training system for strength training, coordination training and sensory training. They were instructed to contract the abdominal muscles and relax the pelvic floor muscles at the same time. This training made the patients recognise the volume needed to first appreciate rectal filling and the maximal tolerable volume needed to elicit an urgent desire.^56^

This study also had several limitations. The basic data of 342 patients were included, and postoperative follow‐up was conducted, but the data sample size was small for model construction. To ensure the stability of the model, only internal cross‐validation was carried out on the dataset, without any external validation. Moreover, this study was a single‐centre study with all hospitalised patients, which could not truly reflect the overall characteristics of LARS patients after colorectal cancer surgery. Despite the regularisation and multicollinearity of variables, some machine‐learning methods, such as the decision tree and random forest algorithms, still pose the risk of over‐fitting; their external applicability needs to be evaluated and verified by prospective cohort studies in the future. Additionally, the models built in this study could be re‐validated and optimised by expanding the amount of data to further improve their prediction performance. In this study, only five machine‐learning methods were used for modelling; therefore, improving the algorithm and using more advanced machine‐learning technology to build a more accurate prediction model could be considered. In the future, preoperative management and consultation of patients at high risk of LARS predicted by the model would be crucial. Medical staff would be able to inform patients of LARS knowledge and nursing methods in detail. Meanwhile, postoperative anal sphincter training would also be essential.

## 5 CONCLUSION

In this study, five machine‐learning classification algorithms—logistic regression, support vector machine, decision tree, random forest and artificial neural network algorithms were applied to model the data from patients with LARS after colorectal cancer surgery, and the fitting effects of the models were compared. The results showed that all exhibited good diagnostic performance, with the decision tree and random forest models having AUCs above 0.85. The logistic regression and decision tree models could visualise the results well and exhibited simplicity in clinical application. We would recommend using the decision tree and logistic regression models to predict the incidence of LARS in postoperative patients with colorectal cancer.

### AUTHOR CONTRIBUTION

**MingJun Huang:** Writing and revising the original draft, Supervision. **Lin Ye**: Writing an original draft, Methodology, Data analysis. **Kexin Yu**: Investigation, Data curation. **Jing Liu:** Investigation, Data curation. **Xiaodong Wang**: Supervision. **Jiping Li**: Supervision.

## FUNDING INFORMATION

This work was supported by research grant from the National Natural Science Foundation of China (Grant number: 72104157).

## CONFLICT OF INTEREST

No conflict of interest was declared by the authors.

## ETHICAL APPROVAL

The study was reviewed and approved by the ethics committees of the West China Hospital, Sichuan University. Written informed consent was obtained from all patients for research purposes.

## CLINICAL TRIAL REGISTRATION NUMBER

This work was registered in Chinese Clinical Trial Registry (Registration number: ChiCTR2100048467).

## Data Availability

All data included in this study are available upon request by contact with the corresponding author.
